# Mutation of the ATPase Domain of MutS Homolog-5 (MSH5) Reveals a Requirement for a Functional MutSγ Complex for All Crossovers in Mammalian Meiosis

**DOI:** 10.1534/g3.119.400074

**Published:** 2019-04-03

**Authors:** Carolyn R. Milano, J. Kim Holloway, Yongwei Zhang, Bo Jin, Cameron Smith, Aviv Bergman, Winfried Edelmann, Paula E. Cohen

**Affiliations:** * Department of Biomedical Sciences and Center for Reproductive Genomics, Cornell University, Ithaca, NY 14853; † Department of Cell Biology, Albert Einstein College of Medicine, Bronx, NY 10461; ‡ Department of Systems and Computational Biology, Albert Einstein College of Medicine, Bronx, NY 10461

**Keywords:** MutS homolog, meiosis, mouse, crossing over, homologous recombination, crossover designation, prophase I

## Abstract

During meiosis, induction of DNA double strand breaks (DSB) leads to recombination between homologous chromosomes, resulting in crossovers (CO) and non-crossovers (NCO). In the mouse, only 10% of DSBs resolve as COs, mostly through a class I pathway dependent on MutSγ (MSH4/ MSH5) and MutLγ (MLH1/MLH3), the latter representing the ultimate marker of these CO events. A second Class II CO pathway accounts for only a few COs, but is not thought to involve MutSγ/ MutLγ, and is instead dependent on MUS81-EME1. For class I events, loading of MutLγ is thought to be dependent on MutSγ, however MutSγ loads very early in prophase I at a frequency that far exceeds the final number of class I COs. Moreover, loss of MutSγ in mouse results in apoptosis before CO formation, preventing the analysis of its CO function. We generated a mutation in the ATP binding domain of *Msh5* (*Msh5^GA^*). While this mutation was not expected to affect MutSγ complex formation, MutSγ foci do not accumulate during prophase I. However, most spermatocytes from *Msh5^GA/GA^* mice progress to late pachynema and beyond, considerably further than meiosis in *Msh5^−/−^* animals. At pachynema, *Msh5^GA/GA^* spermatocytes show persistent DSBs, incomplete homolog pairing, and fail to accumulate MutLγ. Unexpectedly, *Msh5^GA/GA^* diakinesis-staged spermatocytes have no chiasmata at all from any CO pathway, indicating that a functional MutSγ complex is critical for all CO events regardless of their mechanism of generation.

## Introduction

MSH5 (MutS homolog 5) belongs to the DNA mismatch repair (MMR) family of proteins that perform multiple DNA repair activities, most prominently the correction of mispaired bases that result from erroneous DNA replication (Modrich and Lahue 1996). Like other family members, MSH5 acts with a MutS homolog partner, specifically with MSH4, to form the MutSγ heterodimer (Bocker *et al.* 1999). Unlike other MutS heterodimers, MutSγ does not participate in mismatch correction in somatic cells, but instead acts exclusively during meiotic prophase I in budding yeast (Pochart *et al.* 1997), mice (Edelmann *et al.* 1999; de Vries *et al.* 1999; Kneitz *et al.* 2000; Santucci-Darmanin and Paquis-Flucklinger 2003), humans (Bocker *et al.* 1999), plants (Higgins *et al.* 2008), and worms (Zalevsky *et al.* 1999). Indeed, the heterodimer was named MutSγ, with the “γ” referring to “germ cell” (Kolas and Cohen 2004). Importantly, mutation of either MutSγ subunit results in infertility in humans and mice (Edelmann *et al.* 1999; de Vries *et al.* 1999; Kneitz *et al.* 2000; Carlosama *et al.* 2017).

 Prophase I is the defining stage of meiosis, encompassing the unique events that give rise to pairing and equal segregation of homologous chromosomes at the first meiotic division. In early prophase I, homologous chromosomes undergo a physical tethering process known as *synapsis*. Synapsis is mediated by the proteinaceous structure called the Synaptonemal Complex (SC) whose status defines the sub-stages of prophase I: leptonema, zygonema, pachynema, diplonema, and diakinesis. Synapsis is dependent on, and facilitated by, homologous recombination, which is triggered by the formation of DNA double strand breaks (DSBs) by the topoisomerase-like SPO11 protein and its co-factors (Keeney *et al.* 1997; Baudat *et al.* 2000; Romanienko and Camerini-Otero 2000; Keeney 2008; Kim *et al.* 2016; Robert *et al.* 2016a; Robert *et al.* 2016b). DSBs ends undergo resection to reveal 3′ single-strand tails that become coated with the replication protein A (RPA) which protects the potentially fragile ssDNA molecule and impairs secondary structure formation. RPA is gradually replaced by the RecA family members, RAD51 and DMC1, which promote strand invasion to search for homology in opposing chromosomes (Hunter 2015; Gray and Cohen 2016). Strand invasion results in a nascent intermediate known as a displacement loop (D-loop) (Hunter 2015), which may be resolved via multiple distinct, yet overlapping, pathways that result in either a crossover (CO) or a non-crossover (NCO) (Gray and Cohen 2016). In mouse, the majority (approximately 90%) of the 250+ DSBs that form are processed to become NCOs (Cole *et al.* 2014), the remaining 10% being resolved as COs. In yeast, NCOs arise at temporally earlier time points than do the CO repair products (Allers and Lichten 2001; Baudat and de Massy 2007; Jessop and Lichten 2008; Kaur *et al.* 2015).

COs can arise from several pathways down stream of DSB formation, and result in reciprocal exchange of DNA between maternal and paternal homologs, giving rise to the chiasmata that ensure equal segregation of chromosomes at the first meiotic division. Following D-loop formation, a metastable structure known as a single end invasion (SEI) arises, followed by second end capture of the other side of the DSB, to produce a double Holliday Junction (dHJ). These events are promoted through stabilization of the SEI structure by the ZMM group of proteins, of which the MutSγ constituents are members, along with Zip1-4, Mer3, and Spo16 (Lynn *et al.* 2007). Once formed, the dHJ must then be resolved via the action of resolvases which cleave the dHJs to release the recombined homologous chromosomes. In mouse, this is the major Class I crossover pathway, accounting for 90% of all COs, and involves resolution of the dHJ by the MutLγ heterodimer, consisting of the MMR proteins MLH1 and MLH3 (Edelmann *et al.* 1996; Hunter and Borts 1997; Wang *et al.* 1999; Lipkin *et al.* 2002; Svetlanov *et al.* 2008; Nishant *et al.* 2008). In mouse, at least one other CO pathway has been described, known as the class II pathway. Class II events account for fewer than 10% of COs in the mouse and these are dependent on the MUS81-EME1 endonuclease (Oh *et al.* 2008; Holloway *et al.* 2008). This pathway does not involve canonical dHJ formation but instead may resolve a diverse set of repair intermediates that would not ordinarily be strong substrates for the class I machinery.

MutLγ and MutSγ are present on the SC during late pachynema, at a frequency and distribution that resemble class I CO numbers (Santucci-Darmanin and Paquis-Flucklinger 2003). This suggests that, similar to other MMR complexes, MutSγ functions to recruit MutLγ to the SC during pachynema. However, MutSγ foci first appear on meiotic chromosome cores in zygonema, prior to MutLγ localization, and at frequencies that far exceed the final CO tally (approximately 150 foci, or 10-fold higher than the final MutLγ count). These cytogenetic differences in MutSγ/MutLγ appearance suggest additional early functions for MutSγ that are distinct from its interactions with MutLγ. Indeed, the meiotic phenotype of mice lacking components of either complex underscore the temporally distinct roles for each heterodimer. Prophase I spermatocytes from *Mlh1^−/−^* and *Mlh3^−/−^* male mice show normal early progression of meiosis, with cells progressing all the way through prophase I. However, by diplonema, mostly univalent chromosomes are observed in these mutants, with a 90% reduction in chiasmata frequency and loss of spermatocytes prior to the first meiotic division (Edelmann *et al.* 1996; Lipkin *et al.* 2002). By contrast, loss of *Msh4* or *Msh5* results in an earlier loss of prophase I progression, with almost complete failure of homologous synapsis, and cell death prior to pachynema (Edelmann *et al.* 1999; de Vries *et al.* 1999; Kneitz *et al.* 2000). Thus, MutSγ plays an essential role in early events of DSB repair prior to, and distinct from, its proposed role in recruiting MutLγ.

*In vitro* studies have demonstrated that the human and yeast MutSγ heterodimer can bind to D loops, HJs, single-stranded overhangs and other DNA substrates (Snowden *et al.* 2004; Lahiri *et al.* 2018). Binding to junctions enhances stability of these structures, while binding to single-stranded DNA promotes displacement of the overhang that could potentially allow for nucleoprotein filament formation involving, for example, RPA (Lahiri *et al.* 2018). Like all MutS heterodimers, MSH4 and MSH5 each possess an ATPase domain that, upon substrate binding, promotes ADP to ATP exchange and subsequent formation of a sliding clamp that can encircle DNA and translocate away from the binding site, potentially allowing further rounds of MutSγ binding and translocation (Snowden *et al.* 2004, 2008). To explore MSH5 ATPase function *in vivo*, we mutated a highly conserved residue within the P-loop domain of mouse *Msh5* (G to A mutation at residue 596, termed *Msh5*^GA^), which has been shown previously to affect ATP binding by MutS homologs. A similar mutation in *S. cerevisiae* has no effect on the dimerization with its wild-type (WT) MSH4 partner, but reduces crossing over and spore viability (Nishant *et al.* 2010). Based on this study, we anticipated that the G-to-A mutation within the MSH5 ATP binding domain would not affect MutSγ complex formation. Interestingly, although spermatocytes in *Msh5^−/−^* mice fail to progress beyond zygonema, a subset of *Msh5^GA/GA^* spermatocytes escape this fate, progressing through prophase I and entering metaphase I. Thus, this mutant allele allowed for the first time an investigation of the role of MSH5 in crossing over during the prophase I. Interestingly, diakinesis-staged chromosomes from spermatocytes of *Msh5^GA/GA^* mice show exclusively univalent chromosomes and a complete absence of chiasmata, including those residual chiasmata that would presumably arise from the class II CO (MUS81-EME1) pathway. Such residual chiasmata are always observed in mice lacking key class I CO mediators, such as *Mlh1^−/−^* and *Mlh3^−/−^* animals (Edelmann *et al.* 1996; Lipkin *et al.* 2002; Kolas *et al.* 2005). These observations indicate that the ATPase domain of MSH5 is essential for MutSγ activity early in DSB repair, and that mutation of this domain results in disrupted homolog interactions and aberrant DNA repair, leading to a failure to form any COs at the end of prophase I. Thus, loss of a functional MutSγ complex impacts CO formation regardless of the chosen pathway for CO generation.

## MATERIALS AND METHODS

### Generation of Msh5^GA^ mice

The mouse *Msh5* genomic locus was cloned from a P1 mouse ES cell genomic library (Genome Systems) (Edelmann *et al.*, 1999). A 3.6 kb genomic *HindIII* fragment of mouse *Msh5* spanning exons 17-25 was inserted into *pBluescript SK* vector. Positive clones were identified by PCR. The G596A mutation and an analytic *BlpI* restriction site, were generated by site-directed mutagenesis in exon 19. A loxP flanked PGK hygromycin/neomycin cassette was inserted into the *MscI* site in intron 19. The targeting vector was linearized at the single *NotI* site and electroporated into WW6 ES cells. After selection in hygromycin, resistant colonies were isolated and screened by PCR. Positive clones were identified and injected into *C57BL/6J* blastocysts to produce chimeric animals. The PGK hygromycin/neomycin cassette was deleted by Cre-loxP-mediated recombination after mating of chimeric mice to *Zp3Cre* recombinase transgenic females (*C57BL/6J*). F1 offspring were genotyped and heterozygote animals were intercrossed to generate F2 homozygous mutant *Msh5^G^*^A/GA^ mice and appropriate controls. Previously generated *Msh4^-/+^* and *Msh5^-/+^* mice were used for cross breeding studies to provide *Msh5*^-/-^ null mice for comparison (Edelmann *et al.* 1999; Kneitz *et al.* 2000). All *Msh4^+/+^*, *Msh4^−/−^*, *Msh5^+/+^*, *Msh5^+/−^*, *Msh5^−/−^* and *Msh5^GA/GA^* mice used in these studies were backcrossed more than 10 times onto a *C57BL/6J* genetic background. Due to loss of the allele, *Msh5*^-/-^ null mice were not available in the latter half of these studies.

### Genotyping of Msh5^GA^ mice

Reverse transcription-PCR was performed on total RNA isolated from mouse tails with forward primer 5′ – AGACCTGCACTGTGAGATCCG – 3′ (5′-18d-3′) located in exon 16 and reverse primer 5′- TTGGTGGCTACAAAGACGTG-3′ located in exon 22 using the One Tube reverse transcription-PCR reaction kit (Roche) according to the manufacturer’s instructions. The following cycling conditions were used: 30 min at 50° (1 cycle); 2 min at 94°, 45 s at 60°, and 45 s at 68° (37 cycles); and 7 min at 68° (1 cycle). The resulting 480 bp PCR product was subsequently restricted with *Blp*I.

### Care and use of experimental animals

Mice were maintained under strictly controlled conditions of light and temperature, with *ad libitum* access to food and water. All experiments were conducted with prior approval from the Albert Einstein College of Medicine and Cornell Institutional Animal Care and Use Committees. At least six mice per genotype were used for all studies.

### Histological analysis and TUNEL staining of mouse testis

Testes from 12 week- old mice were fixed in Bouin’s fixative for 6 hr at room temperature or 10% formalin overnight at 4°, and then washed in 70% ethanol. Fixed and paraffin-embedded tissues were sectioned at 5 μm. Hematoxylin and eosin (H&E) staining and TUNEL staining and were performed as described previously (Holloway *et al.* 2008, 2010), the latter using Apoptag-peroxidase kit (Millipore).

### Chromosome preparation and spreads

The testes were decapsulated and incubated in hypotonic extraction buffer (HEB; 30 mM Tris, pH 8.2, 50 mM sucrose, 17 mM trisodium citrate dihydrate, 5 mM EDTA, 0.5 mM DTT, and 0.5 mM PMSF) for 1 hr on ice. About three to five millimeters length of seminiferous tubule was transferred into a drop of 20 μl hypotonic sucrose (100 mM, pH 8.2). After adding another drop of 20 μl of sucrose the tubule was macerated and the cell suspension was pipetted up and down for about 3-4 times. Remaining tubule fragments were removed from the cell suspension. Slides were coated with 1% paraformaldehyde containing 0.15% Triton X. 20 μl of the cell suspension were dispersed across the surface of one slide containing a layer of fixative. Slides were transferred to a humid chamber for 1-2 hr at room temperature and then allowed to air dry. Slides were washed three times for 3 min (0.4% Kodak Photo-Flo 200 in water) and air-dried and stored at -80° until use, not longer than 2 weeks.

### Immunofluorescence

The slides were washed in 0.4% Kodak Photo-Flo 200 in PBS and 0.1% Triton X-100 in PBS for 5 min each, blocked for 10 min in 10% antibody dilution buffer (ADB) in PBS (ADB: 3% bovine serum albumin, 0.05% Triton in 1 x PBS) followed by an overnight incubation in primary antibodies (at varying concentrations in ADB; Supplementary Table 1) at room temperature in a humid chamber. Slides were washed as described earlier and incubated for 1 h at 37° in secondary fluorochrome conjugated antibodies in the dark. Primary and secondary antibodies used are listed in Supplementary table 1. All secondary antibodies were raised specifically against Fc fraction, Fab-fraction purified and conjugated to Alexafluor 594, 488, or 647.

### FIJI Image J Macro for SYCP1 & SYCP3 track measurements

An Image J macros was created using the available tools in ImageJ. Images were first converted to TIFF files, with DAPI in blue, SYCP3 in red, and SYCP1 in green. The script used was as follows:

selectWindow(title);

setTool(“freehand”);

run(“Clear Outside”);

run(“Split Channels”);

selectWindow(title +” (red)”);

setAutoThreshold(“RenyiEntropy dark”);

run(“Convert to Mask”);

selectWindow(title+” (green)”);

setAutoThreshold(“RenyiEntropy dark”);

run(“Convert to Mask”);

selectWindow(title+” (red)”);

run(“Skeletonize”);

run(“Analyze Skeleton (2D/3D)”, “prune=[shortest branch] calculate show display”);

selectWindow(title+” (green)”);

run(“Skeletonize”);

run(“Analyze Skeleton (2D/3D)”, “prune=[shortest branch] calculate show display”);

### Spermatocyte diakinesis spread preparations to observe chiasmata

Diakinesis chromosome spreads were prepared as previously described (Holloway *et al.* 2008, 2014). Slides were stained with 20% Giemsa for 2.5 min, washed, air-dried and mounted with Permount.

### Data Availability

All mice, plasmids, and reagents created as part of this study are available on request. Supplemental material available at Figshare: https://doi.org/10.25387/g3.7934312.

## RESULTS

### Generation of Msh5^G596A^ mutant mice

We generated a mouse line bearing a mutation that disrupts the conserved Walker “type A” motif GXXXXGKS/T (G refers to the modified G596 amino acid residue) in the ATPase domain of MSH5, which is important for ATP binding (Figure S1A). The targeting vector introduces a glycine to alanine change at amino acid residue 596 into exon 19 (Figure S1A,B). The mutant allele of this mouse line is designated *Msh5^G596^*^A^ (*Msh5^GA^*) and was predicted to impair ATP binding in the MSH5 subunit. This mutation has previously been shown to preserve interaction with MSH4, allowing for the appropriate assembly of the MutSγ heterodimer (Nishant *et al.* 2010). An additional diagnostic *BlpI* restriction site that does not alter the amino acid sequence was generated in the *Msh5* coding regions that overlaps with the mutation (Figure S1B). Transmission of the mutant *Msh5^GA^* allele was confirmed by PCR genotyping of genomic tail DNA and subsequent restriction of the associated *BlpI* site (Figure S1C,D). Likewise, RT-PCR and subsequent *BlpI* restriction digestion confirmed expression of the mutant transcript in *Msh5^+/GA^* and *Msh5^GA/GA^* mice (Figure S1D). In addition, while homozygous null mice lack all detectable MSH5 protein, the mutant MSH5^GA^ protein signal was found in testis extracts from both *Msh5^GA/+^* and *Msh5^GA/GA^* mice at levels similar to wildtype (WT) mice (Figure S1E). In all subsequent studies, *Msh5^GA/GA^* mutant animals were compared to *Msh5^+/+^* (WT) littermates, as well as *Msh4^−/−^* and/or *Msh5^−/−^* mice (Edelmann *et al.* 1999; Kneitz *et al.* 2000). All alleles of *Msh4* and *Msh5* were maintained on a *C57Bl/6J* background.

### Msh5^GA/GA^ mice exhibit severely impaired meiotic progression, reduced testis size, and no spermatozoa

**Figure 1:**
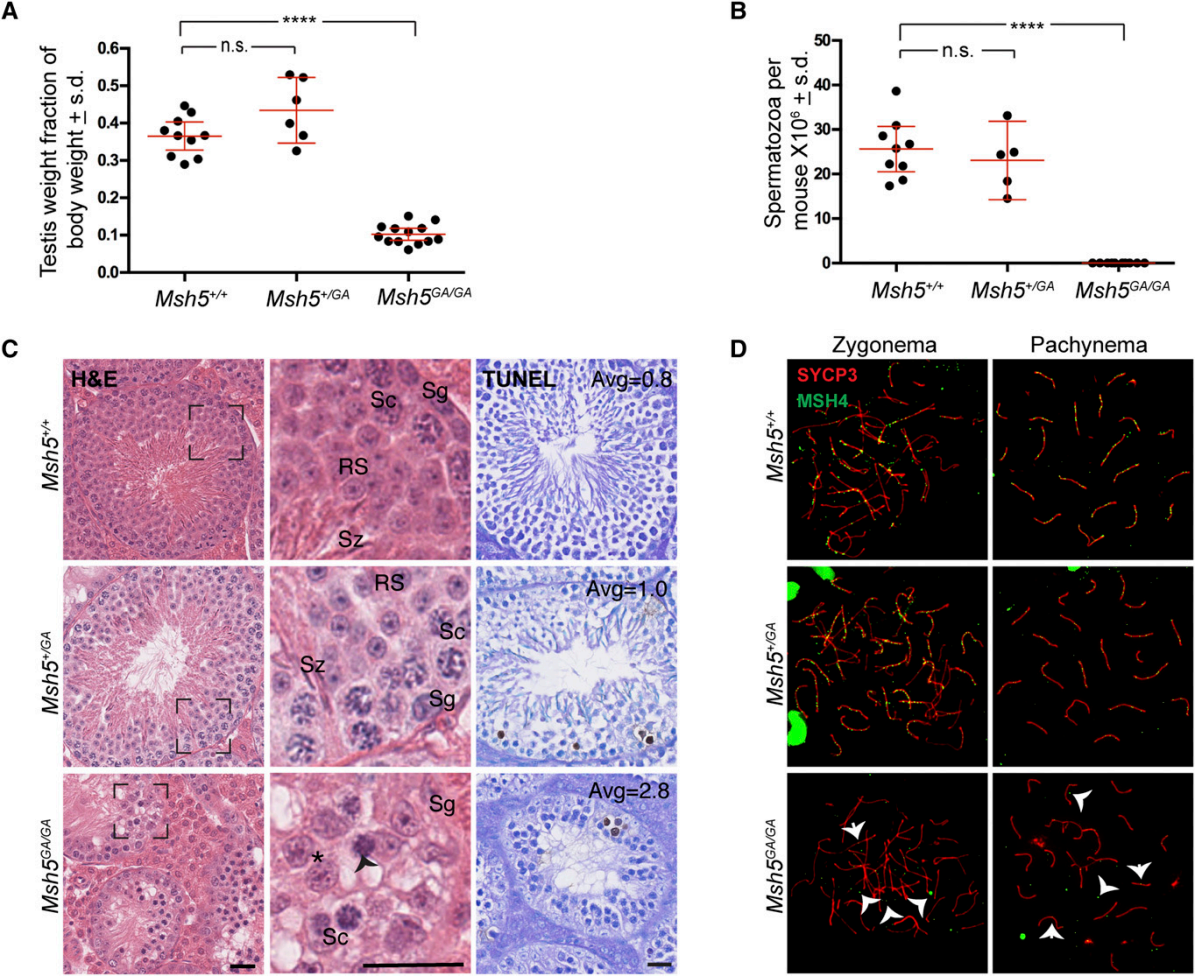
*Msh5^GA/GA^* mice confer an infertility phenotype that is not observed in *Msh5^+/+^* or *Msh5^GA/+^* littermates. (A) Adult testis weights are significantly smaller in *Msh5^GA/GA^* mice compared to *Msh5^+/+^* littermates, while *Msh5^GA/+^* animals are not statistically different to their WT littermates (n = 13, 10 and 6 for *Msh5^GA/GA^*, and *Msh5^+/GA^*, respectively; n.s.- not significant, **** *P* < 0.0001 by unpaired *t*-test with Welch’s correction). Values given are means as a percentage of body weight) ± SD (s.d.). (B) *Msh5^GA/GA^* animals have zero epididymal spermatozoa, while epididymal sperm counts for *Msh5^+/+^* and *Msh5^GA/+^* males are not statistically different from each other (WT compared to *Msh5^GA/GA^* mice: *P* < 0.0001, unpaired *t*-test with Welch’s correction n = 9, n = 10). Values given are means ± SD. (C) Hematoxylin and Eosin staining (two left panels) and TUNEL staining (right panel) of paraffin-embedded testis sections from *Msh5^+/+^*, *Msh5^+/GA^*, and *Msh5^GA/GA^* littermates. WT and heterozygous testes show meiotic and post-meiotic cells whereas *Msh5^GA/GA^* testes are absent of all spermatids and spermatozoa, and apoptotic cells are observed (Sg – spermatogonial, Sc – spermatocytes, RS – Round spermatids, Sz – Spermatozoa, arrow head shows apoptotic cell, asterisk indicate metaphase-like cells; scale bar represents 25 μM). Boxes represent magnified image on the right. TUNEL assay reveals apoptotic cells within seminiferous tubules. The average number of TUNEL-positive cells per tubule are given in each panel. (D) MSH4 (green) and SYCP3 (red) co-localization on chromosome spreads from adult *Msh5^+/+^* and *Msh5^+/GA^* spermatocytes shows MSH4 localization to the SC during zygonema and pachynema, while the association between MSH4 and the SC is largely disrupted in *Msh5^GA/GA^* spermatocytes. Arrow heads in zygotene panel highlight common localization of MSH4, with few foci associated with the SC and frequent foci of varying sizes localized away from the SC. Arrow heads in mutant pachytene panel indicate faint MSH4 signal on or near the SC, suggesting the association between MSH4 and SC is not entirely abolished in the mutants. In all cases, at least 6 mice were studied per genotype, with no fewer than 30 cells per stage being analyzed for each mouse.

Similar to *Msh5^−/−^* and *Msh4^−/−^* mice, *Msh5^GA/GA^* mice are infertile as a result of defects in meiotic prophase I. By contrast, *Msh5^GA/+^* females and males are fertile (not shown), with no change in testis weights in *Msh5^GA/+^* males compared to WT littermates (Figure 1A). Homozygous mutant *Msh5^GA/GA^* males display a 40% reduced testis size compared to their WT littermates (Figure 1A) associated with complete loss of epididymal spermatozoa (Figure 1B). Sperm production in *Msh5^GA/+^* males was similar to that of WT animals (Figure 1B).

Hematoxylin and Eosin (H&E) staining of testis sections from WT adult male mice showed normal cell populations within the seminiferous epithelium, while spermatogenesis was severely disrupted in *Msh5^GA/GA^* testis sections (Figure 1C). Testis sections from *Msh5^GA/GA^* male mice contained Leydig cells, Sertoli cells, and spermatogonia, and spermatocytes, along with a high proportion of TUNEL-positive apoptotic germ cells within the seminiferous tubules (Figure 1C). Most notably, testis sections from *Msh5^GA/GA^* males contained pachytene and post-pachytene spermatocytes (Figure 1C, bottom panels, arrow), including cells that were clearly at metaphase I (Figure 1C, bottom arrows, asterisks). This is in contrast to our previous observations in *Msh5^−/−^* and *Msh4^−/−^* males, in which the majority of the spermatocyte pool is lost at or prior to entry into pachynema (Figure S2A) (Edelmann *et al.* 1999; Kneitz *et al.* 2000). Seminiferous tubules from WT and *Msh5^GA/+^* males have an average of one or less than one TUNEL-positive cell per tubule section, while in *Msh5^GA/GA^* males, TUNEL-positive cell frequencies were higher, at 2.7 TUNEL-positive cells per tubule (Figure S2B). The important difference between the histological appearance of *Msh5^GA/GA^* tubules and that of *Msh5^−/−^* and *Msh4^−/−^* males is the increased progression into pachynema and the appearance of metaphase cells in the tubules of *Msh5^GA/GA^* males.

### A functional ATP binding domain of MSH5 is important for early homolog interactions and complete homolog synapsis

**Figure 2:**
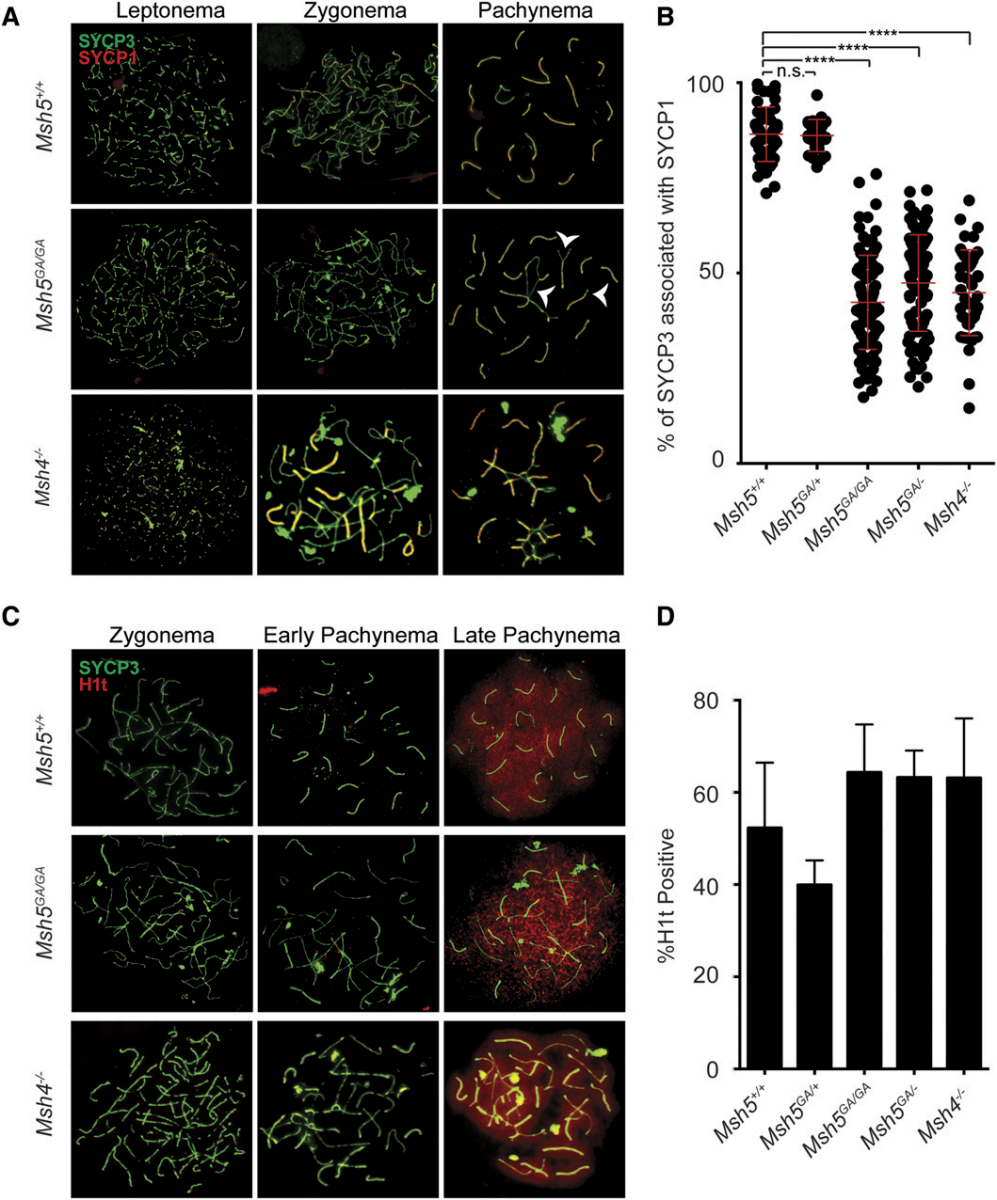
*Msh5^GA/GA^* spermatocytes have inappropriate synapsis between non-homologous chromosomes and progress through mid-pachytene. (A,C) Localization of lateral element SYCP3 (green) with the localization of central element protein SYCP1 (red) in A, or H1t (red) in C. (A) in adult chromosome spreads show the normal progression of synaptonemal complex through prophase I in *Msh5^+/+^* spermatocytes. *Msh5^GA/GA^* spermatocytes have varying degrees of synapsis during mid-prophase I with notable inappropriate synapsis between multi-homologs associations (arrow heads). (B) Synapsis was measured by comparing lengths of total SYCP3 tracks to the total lengths of SYPC1 tracts in each pachytene-like cell using Image J. Percentage of synapsis is calculated per cell by dividing SYCP1 length with SYCP3 length and multiplying by 100. Each point represents a different pachytene-like cell, red overlay lines depict the average ± SD. Synapsis observed in pachytene spermatocytes from *Msh5^+/+^* and *Msh5^GA/+^* animals are not statistically different (unpaired *t*-test with Welch’s correction, *P* = 0.47). *Msh5^GA/GA^*, *Msh5^GA/^*^-^, *and Msh4^−/−^* animals are all found to be significantly different from wild-type (unpaired *t*-test with Welch’s correction, all *P* < 0.0001). (C) Localization of mid-pachytene histone marker, H1t (red) is observed on both *Msh5^+/+^* and *Msh5^GA/GA^* late pachytene spermatocytes, as well as in a subset of spermatocyte from *Msh4^−/−^* animals. (D) % H1t positive prophase I stages are shown for each genotype, with quantitation being limited to late pachytene and diplotene populations. When compared to wild-type, the *Msh5^GA/GA^*, *Msh5^GA/^*^-^, *and Msh4^−/−^* animals all had comparable H1t positive populations across prophase I (unpaired *t*-test with Welch’s correction, *P* = 0.1143, *P* = 0.1714, and *P* = 0.3243 respectively).

To assess progression through prophase I, immunofluorescence (IF) staining was performed on chromosome spreads of spermatocytes from *Msh5^+/+^* and *Msh5^GA/GA^* adult male mice using antibodies against components of the SC, SYCP3 and SYCP1 (Figure 2A). In leptonema, the SC begins to form, with SYCP3 localization appearing in a punctate pattern along asynapsed chromosomes. Such a staining pattern was evident on leptotene chromosome preparations from mice of all genotypes (Figure 2A). Upon entry into zygonema, the transverse filaments and central element of the SC begin to assemble, as shown by the localization of SYCP1 between the chromosome axes on chromosome spreads from both *Msh5^+/+^* and *Msh5^GA/GA^* adult male mice (Figure 2A). By pachynema, when autosomes in *Msh5^+/+^* adult males are now fully synapsed along their entire lengths, the first signs of synapsis failure become evident in *Msh5^GA/GA^* animals.

While WT pachytene cells contain 20 discrete synapsed homologs, spermatocytes from *Msh5^GA/GA^* animals show variable degrees of synapsis, coupled with frequent occurrences of inappropriate synapsis between more than two chromosome partners (Figure 2A, arrowheads), indicating non-homologous synapsis events between multiple chromosomes, but also some occurrences of apparently normal homolog synapsis. Thus, in order to stage these spermatocytes from *Msh5^GA/GA^* animals, we defined certain criteria for each prophase I substage. Zygotene and diplotene spermatocytes, which often look similar, were distinguished based on the length of the SC (longer in zygonema), differences in telomeric ends of the chromosomes (more bulbous in diplonema), and by H1t localization (see below). A “pachytene-like” stage was defined as having ≥4 discrete synapsed chromosome pairs, either wholly or partially, along with a more condensed SC appearance across all chromosomes. Using these criteria, we observed many cells in a pachytene-like stage, and beyond, in *Msh5^GA/GA^* animals. The aberrant synapsis phenotype observed in *Msh5^GA/GA^* spermatocytes range in severity, with some pachytene-like cells showing synapsis defects across the majority of homolog pairs, while other pachytene-like cells showed defects among a few homolog pairs.

Utilizing Image J software, we obtained quantitative measurements of synapsis across our mouse model. For each cell, we measured the total track length of SYCP3 signal and compared it to the total track length of SYPC1 to obtain the percent synapsis (SYCP1/SYCP3 X 100). For this analysis, we used *Msh4^−/−^* mice as a comparison with *Msh5^GA/GA^* males because the original reports suggested slightly higher levels of synapsis than observed in *Msh5^−/−^* mice, and because *Msh5^−/−^* mice are no longer available. Since MSH4 and MSH5 always act as a heterodimer, *Msh4^−/−^* mice reflect overall MutSγ function. Previous descriptions of *Msh4^−/−^* males indicated no pachytene entry, an observation that was based on the 20 independently synapsed homologs as defined by WT pachytene. In the current study however, we defined pachytene-like as >4 synapsed or partially synapsed chromosomes. Under these criteria, we observe pachytene-like cells in both *Msh4^−/−^* males and in *Msh5^GA/GA^* males.

The average synapsis in WT spermatocytes during pachynema, remembering the XY chromosome pair in males is only synapsed at the autosomal region, is 86.5 ± 7.2% (Figure 2B) with *Msh5^GA/+^* spermatocytes showing similar synapsis rates at 86.3 ± 4.2% (Figure 2B). Overall there is a remarkable degree of synapsis in *Msh5^GA/GA^* animals, with spermatocytes exhibiting an average of 43.2 ± 12.4%, and some cells achieving up to 76% of chromosome axes. By contrast, synapsis in *Msh5^−/−^* animals is less than 5% in two previous reports (Edelmann *et al.* 1999; de Vries *et al.* 1999). The level of synapsis in *Msh5^GA^*^/-^ males is comparable to that of *Msh5^GA/GA^* males, at 47.4 ± 12.7% synapsis. Synapsis in *Msh4^−/−^* males was slightly lower than *Msh5^GA/GA^* males, at 44.9 ± 11.3% synapsis (Figure 2B). Importantly, synapsis in *Msh5^GA/^*^-^ spermatocytes is similar to that seen in *Msh5^GA/GA^* homozygous mutant animals, while synapsis in *Msh5^GA/+^* spermatocytes is similar to WT, indicating that the *Msh5^GA^* allele is recessive and not causing a dominant negative effect.

To further assess the degree of synapsis in different mice, the number of independently synapsed homologs were counted in each pachytene-like cell from *Msh5^GA/GA^*, *Msh5^GA/^*^-^, and *Msh4^−/−^* males (Figure S3). In none of these cases are cells from mutant testes able to achieve a wild-type pachytene configuration of 20 independently synapsed homologs. While some *Msh4^−/−^* pachytene cells were only able to achieve as many as 12 independently synapsed homologs, only 4.8% of the pachytene-like population had more than 10 independently synapsed homologs. The degree of synapsis is significantly greater in *Msh5^GA/GA^* and *Msh5^GA/^*^-^ spermatocytes, with instances of cells achieving up to 15 independently synapsed homologs occurring in each genotype, and *Msh5^GA/GA^* having 5.6% homologs having more than 10 independently synapsed homologs and *Msh5^GA/^*^-^ having 11.6% homologs having more than 10 independently synapsed homologs (Figure S3). Thus, we observe a greater degree of synapsis in spermatocytes from *Msh5^GA/GA^* or *Msh5^GA/^*^-^ males compared to that of *Msh4^−/−^* cells suggesting that the presence of the mutant MSH5^GA^ protein allows for more proficient early homolog pairing and progression through later stages of prophase I. Thus, homolog pairing and/or synapsis initiation/progression does not rely on a fully functional MutSγ heterodimer.

The histone marker, H1t, allows for differentiation of pachytene cells into “early” and “late”, since H1t only associates with the latter population (Wiltshire *et al.* 1995). Moreover, H1t positive staining allows for differentiating between zygotene and diplotene-like cells in *Msh5^GA/GA^* males. Despite the incomplete synapsis and inappropriate synapsis between multiple chromosomes in spermatocytes from *Msh5^GA/GA^* animals, these cells are competent to achieve a mid-pachytene-like stage of meiosis, at least as assessed by acquisition of H1t signal (Figure 2C). Synapsis mutants (*Msh5^GA/GA^*, *Msh5^GA/^*^-^, *Msh4^−/−^*) do not achieve the normal 20 independently synapsed homologs as observed in WT. However, the localization of H1t to these mutants suggests that they achieve a pachytene-like stage. To compare prophase I populations across genotypes, we looked at the total number of H1t-positive cells in prophase I (Figure 2D). Across all prophase I cells in WT males, we observe that 52.3 ± 14.1% of cells are H1t-positive. Surprisingly, our mutant animals gave values similar to WT: in *Msh5^GA/GA^* animals we observe a 64.4 ± 10.1% H1t positive prophase I population, in *Msh4^−/−^* 63.2 ± 12.9%; *Msh5^-/GA^* 63.3 ± 5.8%. The *Msh5^+/GA^* spermatocytes are the only population for which we observed a lower, albeit not statistically different H1t-positive prophase I pool of 40.0 ± 5.3%. Overall, we observed a comparable prophase I progression in *Msh5^GA/GA^* mutant spermatocytes and in *Msh4^−/−^* spermatocytes, although the degree of synapsis observed in these mutants is markedly different.

### MutSγ association with the synaptonemal complex is drastically reduced in spermatocytes From Msh5^GA/GA^ males

In WT mice, MSH4 and MSH5 localize on chromosome cores of the SC from zygonema through pachynema, with approximately 200 foci in zygonema, reducing progressively through until late pachynema (Kneitz *et al.* 2000). We investigated whether MutSγ localization on SCs was affected by loss of a functional ATP binding domain within MSH5. To this end, chromosome spreads from *Msh5^+/+^*, *Msh5^+/GA^*, and *Msh5^GA/GA^* male mice were subjected to IF staining using antibodies against MSH4 and SYCP3. MSH4 localization in early prophase I cells looks comparable between *Msh5^+/+^* and *Msh5^+/GA^* adult males, with abundant foci associated with early SC structures in zygonema (Figure 1D). Interestingly, in spermatocytes from *Msh5^GA/GA^* males, there appears to be a dramatically decreased association of MSH4 to the SC and an observable increase in MSH4 foci not associated with the SC in zygotene and pachytene nuclei (Figure 1D). Overall the intensity of MSH4 staining in zygotene and pachytene spermatocytes from *Msh5^GA/GA^* males is lower than that of WT littermates, although some foci are clearly associated with the SC at both zygonema and pachynema (Figure 1D, arrows). Further examples of MSH4 staining at this stage are provided in Figure S4 which provide additional evidence of a broader but fainter distribution of MSH4 signal in spermatocytes from *Msh5^GA/GA^* males.

### The ATP binding domain of MSH5 is essential for timely progression of DSB repair events

**Figure 3:**
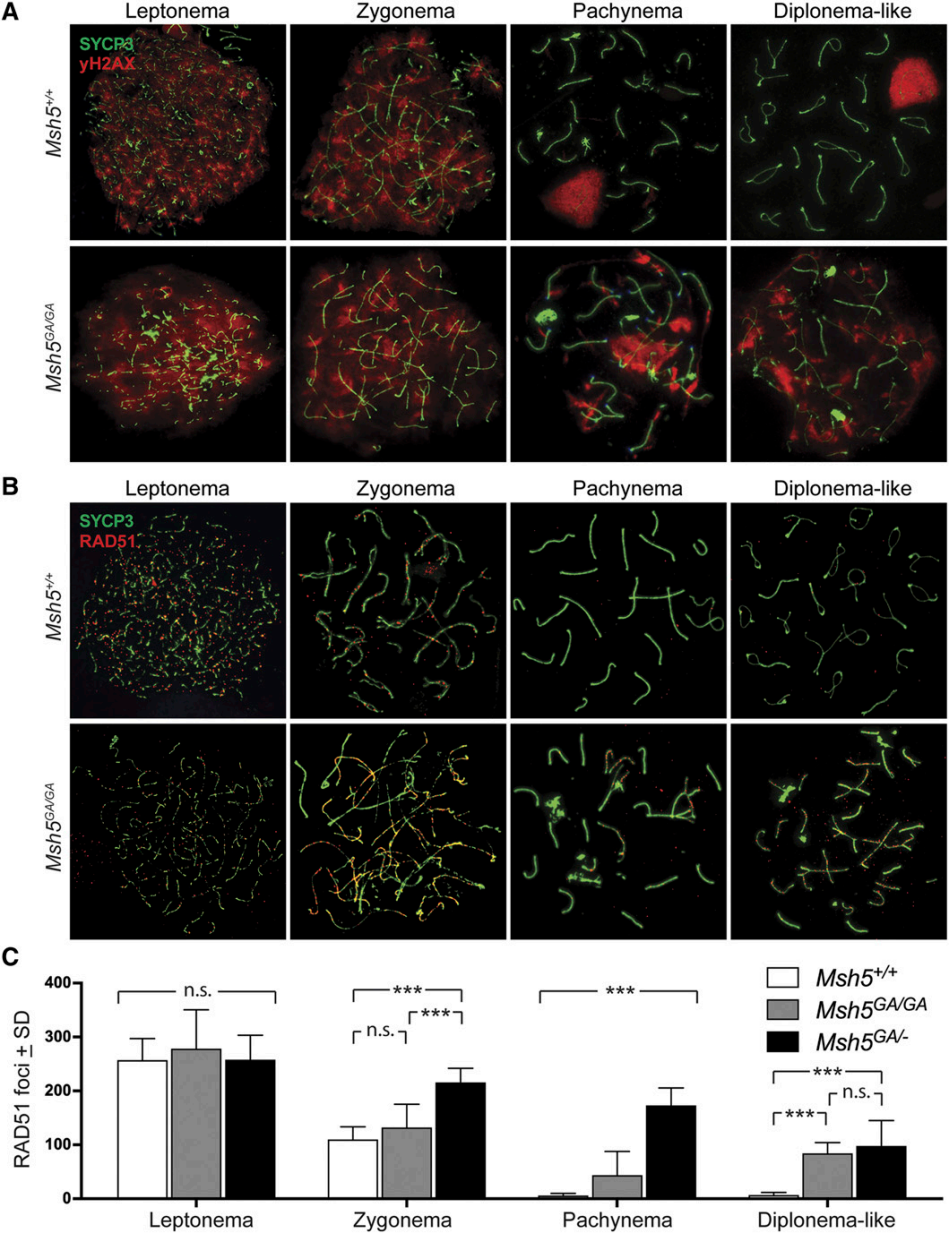
DNA damage persists in *Msh5^GA/GA^* spermatocytes throughout prophase I. (A) Immunofluorescent staining of γH2AX (red) on chromosome spreads of *Msh5^+/+^* and *Msh5^GA/GA^* littermates. (B) DNA repair marker RAD51 (red) on *Msh5^+/+^* and *Msh5^GA/GA^* chromosome spreads persists throughout prophase I. (C) Quantitation of RAD51 foci associated with the SC of chromosome spreads during leptonema (n = 13 and 14, respectively, for *Msh5^+/+^* and *Msh5^GA/GA^* males; *P* = 0.88 by Mann-Whitney), zygonema (n= 30 and 29, respectively; *P* = 0.14 by Mann-Whitney), pachynema (n = 26 and 35, respectively; *P* < 0.0001 by Mann-Whitney) and diplonema (n = 11 each; *P* < 0.0001 by Mann-Whitney). RAD51 counts were also assessed for *Msh5^GA/^*^-^ males at each stage and were significantly different to that of *Msh5^GA/GA^* males at zygonema and pachynema (*P* < 0.0001 by Mann-Whitney), and statistically different to *Msh5^+/+^* males at all stages (*P* < 0.0001 by Mann-Whitney) except leptonema (*P* = 0.86 by Mann-Whitney).

To assess progression of DSB repair through prophase I, IF was performed on chromosome spreads from *Msh5^+/+^*, *Msh5^GA/GA^*, and *Msh5^+/GA^* adult littermates using antibodies against γH2AX, a phosphorylated histone variant that marks sites of DSB (Figure 3A). Spermatocytes from *Msh5^+/+^* animals show a strong γH2AX signal during leptonema and zygonema of prophase I indicating normal induction of DSBs, with loss of the γH2AX signal at pachynema signaling progression of DSB repair (Figure 3A). As expected, the γH2AX signal is intensified on the sex chromosomes at pachynema, a phenomenon that is not related to DSB formation (Turner *et al.* 2004); (Figure 3A, top row). Spermatocytes from *Msh5^GA/GA^* animals show a similarly strong γH2AX signal during leptonema and zygonema, indicating DSBs are induced at the expected time. Unlike in *Msh5^+/+^* cells, however, γH2AX signal is retained on autosomes throughout prophase I in *Msh5^GA/GA^* cells, indicating persistent DNA damage (Figure 3A, bottom row).

During DSB repair, one of the earliest common intermediate steps involves strand invasion and homology search, which is mediated by the RecA homologs, RAD51 and DMC1. MutSγ has been suggested to participate in stabilization of these strand invasion events *in vitro* (Snowden *et al.* 2004). During leptonema in WT spermatocytes, RAD51 foci are observed on axial elements of the SC in high numbers (Figure 3B,C), and similar numbers of RAD51 foci are observed on leptotene spreads from *Msh5^GA/GA^* spermatocytes. As WT cells progress from zygonema to pachynema, RAD51 foci numbers drop dramatically, reflecting the repair of DSBs.

The RAD51 focus numbers in *Msh5^GA/GA^* and *Msh5^GA/^*^-^ spermatocytes remain significantly elevated above that of WT spermatocytes throughout prophase I (*P* < 0.0001, Figure 3C). Interestingly, the RAD51 focus counts at zygonema and pachynema are significantly higher in *Msh5^GA/^*^-^ spermatocytes than in homozygous mutant *Msh5^GA/GA^* spermatocytes (*P* < 0.0001, Figure 3C), indicating more DSB repair activity during this stage in the presence of only one copy of ATPase defective *Msh5*. At diplonema, WT spermatocytes have lost all RAD51 foci, but these foci remain significantly higher in *Msh5^GA/GA^* and *Msh5^GA/^*^-^ spermatocytes, albeit at lower frequency to that seen in pachynema (*P* < 0.0001). At this stage, RAD51 counts in *Msh5^GA/GA^* and *Msh5^GA/^*^-^ spermatocytes are not statistically different from each other. Importantly, spermatocytes from *Msh5^GA/+^* males are similar to WT with few abnormalities and normal dynamics of RAD51 loss (Figure 3C, S3B).

Taken together, these data demonstrate the presence of the ATP binding-defective MSH5^GA^ protein is critical for normal progression of DSB repair. Alternatively, it is possible that the high rate of RAD51 foci observed at pachynema in *Msh5^GA/GA^* males results from additional induction of DSBs through prophase I, but the current tools preclude our ability to differentiate between these two options. Importantly, the presence of only one GA allele on a WT background (*Msh5^GA/+^* males) results in normal temporal dynamics of RAD51 loss, while the presence of one GA allele on a null background (*Msh5^GA/^*^-^ males) results in a significantly more delayed processing of DSBs, as characterized by RAD51 accumulation and loss. These observations argue strongly against a dominant negative effect of the GA point mutation.

### An intact MSH5 ATP binding domain is essential for formation of all classes of crossover

**Figure 4:**
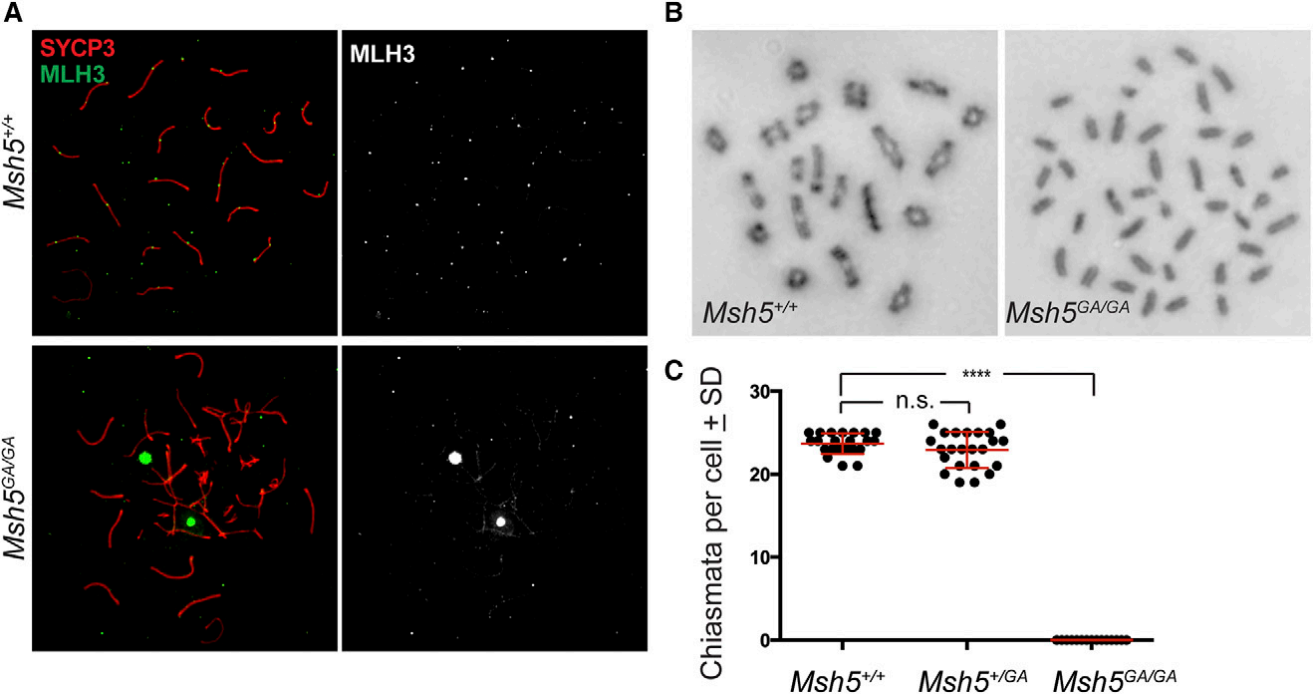
No crossovers form in *Msh5^GA/GA^* spermatocytes. (A) Immunofluorescence staining of MLH3 (green) on SYCP3-stained SC cores (red) in adult pachytene *Msh5^+/+^* and *Msh5^GA/GA^* spermatocytes show localization of MLH3 to SC as expected in wild type and no MLH3 localization to the SC in *Msh5^GA/GA^* males. (B) Giemsa staining of diakinesis preparations from *Msh5^+/+^* and *Msh5^GA/GA^* litter mates showing normal chiasmata in wild type cells, with 20 bivalent chromosomes, and all univalent chromosomes in spermatocytes from *Msh5^GA/GA^* males. (C) Chiasmata counts for *Msh5^+/+^* (n = 22), *Msh5^+/GA^* (n = 23), and *Msh5^GA/GA^* (n = 15) littermates (*P* < 0.0001, unpaired *t*-test). Each circle symbol represents a different cell, while the red overlay lines depict the average ± SD.

MutSγ recruits the MutLγ complex during pachynema as part of a canonical class I CO machinery. IF staining using antibodies against MLH3 was compared across genotypes (Figure 4A). In WT and *Msh5^GA/+^* mice during pachynema, MLH3 appears on chromosome cores at a frequency that correlates with final class I CO numbers (Figure 4A, top row), but is absent in spermatocytes from *Mlh3^−/−^* males (Figure S5B). In pachytene-like spermatocytes from *Msh5^GA/GA^* males, MLH3 foci do not form on chromosome cores (Figure 4A). Occasional very faint signal was observed throughout the chromatin, as well on the SC cores, when the microscope intensity gain is increased, but it was not possible to obtain reliable images depicting this weak signal. Nonetheless, such staining was never observed in chromosome spread preparations from *Mlh3^−/−^* males, suggesting that this weak staining might be specific for MLH3 protein (Figure S5B). Given the diffuse and faint nature of this staining, it cannot be determined if this MLH3 signal is associated with sites of DSB repair. Thus, a fully functional MSH5 protein is required for appropriate association of the MutLγ with the synaptonemal complex and establishment of nascent class I CO sites.

To assess crossing over across the genome, diakinesis spreads were prepared to assess chiasmata formation (Holloway *et al.* 2010). In WT males, each bivalent chromosome pair had at least one chiasmata (Figure 4B). Since a small number of spermatocytes from *Msh5^GA/GA^* males are capable of progressing into diakinesis, we were able to count chiasmata in these homozygous mutant mice (Figure 4B,C). Unexpectedly, diakinesis-staged cells from *Msh5^GA/GA^* males displayed exclusively univalent chromosomes and did not form any chiasmata (Figure 4B,C). Thus, normal MSH5 ATP processing is essential for all crossover formation in mammals. Such analysis has not been possible in *Msh5^−/−^* males because spermatocytes from these mice fail to reach diakinesis, and die predominantly in zygonema.

## Discussion

The data presented herein demonstrate that intact MutSγ function is required for normal prophase I progression in male meiosis. Importantly, this work is the first to show a definitive requirement for an intact MutSγ heterodimer in crossing over in the mouse and, unexpectedly, that MutSγ is critical for all crossovers regardless of their route of generation from DSB precursors. These observations were made possible by the fact that the mutation in the MSH5 ATP binding domain can allow for limited progression through to the end of prophase I, whereas most spermatocytes from *Msh5^−/−^* mice die prior to pachynema (Edelmann *et al.* 1999; de Vries *et al.* 1999). Mutation of the ATP binding domain within *Msh5* results in normal DSB induction but prolonged RAD51 installation on chromosome cores, either due to delayed DSB repair or due to extended DSB initiation through prophase I. As a result, we demonstrate a greater degree of synapsis observed in spermatocytes from *Msh5^GA/GA^* or *Msh5^GA/^*^-^ males compared to that of *Msh4^−/−^* cells, suggesting that the presence of the MSH5^GA^ protein allows for more proficient early homolog pairing, or that the SC is established more robustly in the presence of defective MutSγ heterodimer than in the complete absence of any heterodimer.

Data presented herein also demonstrate altered distribution of MutSγ throughout the nucleus of *Msh5^GA/GA^* prophase I spermatocytes, with significant localization off the SC, and a reduction in overall MSH4 signal on chromosome cores. These results indicate that the MSH5 ATP binding domain is essential for the recruitment and retention of MutSγ on SC cores from zygonema through until pachynema. Loss of ATP binding in *Msh5^GA/GA^* mutants is predicted to result in a clamp protein that is unable to slide along DNA, and thus is unable to allow successive rounds of MutSγ loading. In our *Msh5^GA/GA^* mutants we see a dramatic reduction in MSH4 signal along chromosome cores, suggesting either minimal loading of MutSγ complex onto the DNA and/or enhanced (but not complete) degradation of the complex. Thus, the low amount of MSH5^GA^-MSH4 heterodimer that can associate with the SC may still provide some stabilization between homologs, allowing for small amounts of synapsis in *Msh5^GA/GA^* animals. However, without the MSH5 ATP domain, the normal function of MutSγ in SC establishment and/or DSB repair processing is abolished. Taken together, we conclude that early DSB repair events and synapsis are perturbed in our *Msh5^GA/GA^* mutants, but that some progression remains possible. Importantly, these observations suggest that the ATP binding domains of both MutSγ subunits must be intact in order to facilitate a complete repertoire of MutSγ functions, which is not surprising given the fact that MSH5 has been shown to bind ATP with a higher affinity than MSH4 (Snowden *et al.* 2008).

In the mouse, MutSγ accumulation on SCs in zygonema is in excess of the final number of MutLγ foci, but the two heterodimeric complexes are shown to localize at similar frequencies by late pachynema, albeit with number of MutSγ foci remaining slightly higher than MutLγ (Novak *et al.* 2001; Santucci-Darmanin and Paquis-Flucklinger 2003). The earlier and more abundant localization of MutSγ in zygonema implies that MutLγ is recruited to only a subset of MutSγ sites upon entry into pachynema, with the remaining sites that fail to accumulate MutLγ presumably being processed to become NCO events via other repair pathways. Thus, the higher numbers of MutSγ foci in zygotene and early pachytene mouse spermatocytes, together with the earlier loss of spermatocytes in *Msh5^−/−^* animals compared to *Mlh3^−/−^* or *Mlh1^−/−^* mice, implies a role for MSH4 and MSH5 in DSB processing at an early intermediate stage for multiple repair pathways. Such a possibility is supported by our data showing that diakinesis preps from *Msh5^GA/GA^* spermatocytes display no chiasmata (Figure 4), which indicates that a functional MutSγ complex is essential for all CO, acting at a stage that is upstream of both class I and class II CO designation, and thus may be a common intermediate for all CO pathways early in prophase I. Indeed, both Class I and Class II crossovers arise from a common DNA repair intermediate structure downstream of RAD51/DMC1 activity.

**Figure 5:**
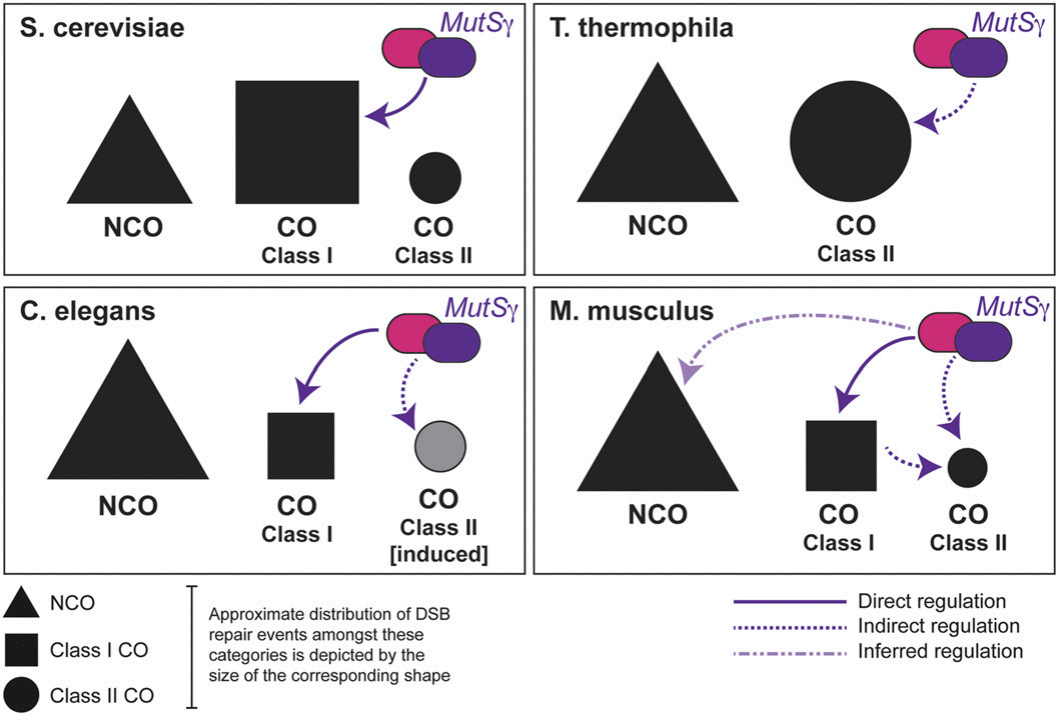
Model for MutSγ function during meiosis in different eukaryotes. Model of MutSγ role in CO establishment across eukaryotes. In *S. cerevisiae*, MutSy functions specifically in promoting Class I COs. In *T. thermophila*, where class I COs are absent, MutSγ functions to promote class II COs. In *C. elegans*, MutSγ functions to drive COs, all of which are class I COs under normal conditions. In conditions of induced DNA damage, MutSγ may also promote the extraneous class II COs that arise. In *M. musculus*, as shown by this study, MutSγ functions is essential to all CO repair, and is likely associated with a subset of non CO DNA repair events. See text for further details.

While the class II CO pathway, which in mice involves MUS81-EME1 (Holloway *et al.* 2008; Schwartz and Heyer 2011), is not traditionally viewed to be dependent on the ZMM class of proteins, and persists in mice lacking either *Mlh1* or *Mlh3*, our data indicate that a functional MSH5 protein is required to promote both classes of CO. Conversely, while we briefly considered the possibility that the mutant MutSγ complex may bind irreversibly to DSB repair intermediates that might otherwise have been processed via the Class II pathway, thus blocking the recruitment of appropriate class II repair factors, this does not appear to be the case since severely reduced MSH4 signal is observed on the SC, while no meiotic phenotype is observed in *Msh5^GA/+^* males, arguing against a dominant negative effect. Thus, loss of appropriate loading of MutSγ on the SC is sufficient to prevent any CO processing, regardless of the pathway of repair. This argues against current dogma that states that ZMM proteins, of which MSH4 and MSH5 are family members, do not operate outside of the class I machinery. While our current data do not currently provide a mechanism by which MutSγ can orchestrate both CO pathways in mammals, studies from other organisms provide interesting insight into potential mechanisms. In *Tetrahymena thermophila*, for example, which has no SC, COs are exclusively of the class II variety, requiring Mus81-Mms4, but not the canonical ZMM family. Despite the absence of class I CO events, MSH4 and MSH5 are essential for appropriate CO levels in this species, leading to the conclusion that these proteins function outside (or upstream) of the canonical class I CO pathway (Shodhan *et al.* 2014) (Figure 5).

In SC-bearing organisms, where class I and class II CO events occur in tandem to differing degrees, ZMM proteins appear to function exclusively in the metabolism of the former class of COs. In *S. cerevisiae*, CO assignment occurs prior to SC assembly, and the number of MSH5 foci observed in this species corresponds well with the final tally of class I COs (Agarwal and Roeder 2000) (Figure 5). However, this does not appear to be the case for organisms such as *C. elegans*, in which only class I COs occur. Yokoo *et al.* have proposed that the installation of MSH-5 in worms represents a “CO licensing” stage during which the protein initially accumulates at a supernumerary frequency along the chromosome cores (Yokoo *et al.* 2012). These foci then diminish in number as the cell progresses through pachynema in *C. elegans*, accumulating the pro-crossover factor COSA-1 only once the final number of class I events is achieved. Thus, the final appearance of COSA-1 and MSH-5 bound foci at six sites across the worm genome represents the final “designation” of presumptive class I CO sites (Yokoo *et al.* 2012) (Figure 5).

In the mouse, the same excessive number of MutSγ foci appear somewhat earlier in prophase I, at or soon after the completion of the axial elements in early zygonema, and these too get pared down through zygonema and pachynema coincident with the progression of CO designation. Loss of the entire MSH5 protein results in a failure to accumulate MutSγ or to complete synapsis in zygonema, resulting in cell death prior to pachynema or, at the very most, aberrant progression through pachynema (Edelmann *et al.* 1999; Kneitz *et al.* 2000). Thus in the mouse, CO licensing is tightly linked to appropriate synapsis and may reflect the requirement for distinct rearrangements in SC architecture by the MutSγ complex, similar to that proposed for *C. elegans* (Pattabiraman *et al.* 2017). However, in the current study, we find that loss of a functional ATPase domain in one component of MutSγ, MSH5, allows for partial synapsis implying that any structural changes to the SC can be orchestrated in the absence of full ATPase activity of the MutSγ complex. Under such circumstances, all COs are lost, regardless of their final pathway of biogenesis. Thus, CO processing through both the class I and class II pathways is dependent on a fully functional MutSγ heterodimer, but may not be dependent on any SC changes induced by MutSγ in zygonema.

Our data suggest either that functional activity of class II machinery depends on the presence and processing of class I COs (an indirect requirement perhaps involving more discrete localized changes in the SC state at the DSB site), or that loading of class II pathway mediators requires the presence of MutSγ at these sites (a direct requirement for loading of MutSγ prior to recruitment of class II repair factors). In either case, this would infer that MutSγ is required for CO licensing for both pathways and/or lies upstream of the licensing decision. This is not surprising given that, in the mouse, no fewer than 60% of the DSB sites become loaded with MutSγ (or 150 out of 250), and only a minor fraction of these licensed sites (approximately 20%) will become COs of the class I or class II variety (Kneitz *et al.* 2000; Cole *et al.* 2012). Thus, there is an over-abundance of available sites for crossing over and, suggesting that MutSγ loads as efficiently onto NCO-destined DSB repair intermediates as it does onto CO-destined DSB repair intermediates. Though the implication of this promiscuous MutSγ binding is not yet understood, it suggests that, while CO licensing in worms is achieved by MSH-5 association, this may not be the case in the mouse since MutSγ association with DSB repair intermediates appears to be more promiscuous than in worm and yeast.

Taken together, our analysis of a point mutant mouse for *Msh5* has allowed us for the first time to explore late prophase I roles for MSH5 in DSB repair and homologous recombination. Our observations demonstrate that the large number of MutSγ sites found in early prophase I may serve as intermediates for both class I and class II CO events, and indeed for NCO events. Moreover, unlike the situation in yeast, the early loading of MutSγ in mouse spermatocytes suggests progressive NCO formation through prophase I. Given that MutLγ is restricted to class I CO events, these data suggest a functional distinction between the roles of MutSγ and MutLγ in DSB repair during mammalian meiosis, and open the door for additional roles for MutSγ in orchestrating/overseeing DSB repair in the mammalian germline. In light of the role of other heterodimeric MutS complexes in recruiting a diverse array of repair pathways, we envisage that MutSγ serves a similar purpose in the context of DSB repair during mammalian meiosis, serving as a point of dialog between multiple repair pathways to achieve genome stability.

## References

[bib1] Agarwal S. Roeder G. S., 2000 Zip3 provides a link between recombination enzymes and synaptonemal complex proteins. Cell 102: 245–255. 10.1016/S0092-8674(00)00029-5 10943844

[bib2] Allers T. Lichten M., 2001 Differential timing and control of noncrossover and crossover recombination during meiosis. Cell 106: 47–57. 10.1016/S0092-8674(01)00416-0 11461701

[bib3] Baudat F. Manova K. Yuen J. P. Jasin M. Keeney S., 2000 Chromosome synapsis defects and sexually dimorphic meiotic progression in mice lacking Spo11. Mol. Cell 6: 989–998. 10.1016/S1097-2765(00)00098-8 11106739

[bib4] Baudat F. de Massy B., 2007 Regulating double-stranded DNA break repair towards crossover or non-crossover during mammalian meiosis. Chromosome Research 5: 565–577. 10.1007/s10577-007-1140-3 17674146

[bib5] Bocker T. Barusevicius A. Snowden T. Rasio D. Guerrette S., 1999 hMSH5: a human MutS homologue that forms a novel heterodimer with hMSH4 and is expressed during spermatogenesis. Cancer Res. 59: 816–822. 10029069

[bib6] Carlosama C. El Zaiat M. Patiño L. C. Mateus H. E. Veitia R. A., 2017 A homozygous donor splice-site mutation in the meiotic gene MSH4 causes primary ovarian insufficiency. Hum. Mol. Genet. 26: 3161–3166. 10.1093/hmg/ddx199 28541421

[bib7] Cole F. Baudat F. Grey C. Keeney S. de Massy B., 2014 Mouse tetrad analysis provides insights into recombination mechanisms and hotspot evolutionary dynamics. Nat. Genet. 46: 1072–1080. 10.1038/ng.3068 25151354 PMC4207963

[bib8] Cole F. Kauppi L. Lange J. Roig I. Wang R., 2012 Homeostatic control of recombination is implemented progressively in mouse meiosis. Nat. Cell Biol. 14: 424–430. 10.1038/ncb2451 22388890 PMC3319518

[bib46] de Vries S. S. Baart E. B. Dekker M. Siezen A. de Rooij D. G., 1999 Mouse MutS-like protein Msh5 is required for proper chromosome synapsis in male and female meiosis. Genes Dev. 13: 523–531. 10.1101/gad.13.5.523 10072381 PMC316502

[bib9] Edelmann W. Cohen P. E. Kane M. Lau K. Morrow B., 1996 Meiotic pachytene arrest in MLH1-deficient mice. Cell 85: 1125–1134. 10.1016/S0092-8674(00)81312-4 8674118

[bib10] Edelmann W. Cohen P. E. Kneitz B. Winand N. Lia M., 1999 Mammalian MutS homologue 5 is required for chromosome pairing in meiosis. Nat. Genet. 21: 123–127. 10.1038/5075 9916805

[bib11] Gray S. Cohen P. E., 2016 Control of meiotic crossovers: from double-strand break formation to designation. Annu. Rev. Genet. 50: 175–210. 10.1146/annurev-genet-120215-035111 27648641 PMC5319444

[bib12] Higgins J. D. Vignard J. Mercier R. Pugh A. G. Franklin F. C. H., 2008 AtMSH5 partners AtMSH4 in the class I meiotic crossover pathway in Arabidopsis thaliana, but is not required for synapsis. Plant J. 55: 28–39. 10.1111/j.1365-313X.2008.03470.x 18318687

[bib13] Holloway J. K. Booth J. Edelmann W. McGowan C. H. Cohen P. E., 2008 MUS81 generates a subset of MLH1–MLH3-independent crossovers in mammalian meiosis. PLoS Genet. 4: e1000186 10.1371/journal.pgen.1000186 18787696 PMC2525838

[bib14] Holloway J. K. Morelli M. A. Borst P. L. Cohen P. E., 2010 Mammalian BLM helicase is critical for integrating multiple pathways of meiotic recombination. J. Cell Biol. 188: 779–789. 10.1083/jcb.200909048 20308424 PMC2845075

[bib15] Holloway J. K. Sun X. Yokoo R. Villeneuve A. M. Cohen P. E., 2014 Mammalian CNTD1 is critical for meiotic crossover maturation and deselection of excess precrossover sites. J. Cell Biol. 205: 633–641. 10.1083/jcb.201401122 24891606 PMC4050721

[bib16] Hunter N., 2015 Meiotic recombination: the essence of heredity. Cold Spring Harb. Perspect. Biol. 7: a016618 10.1101/cshperspect.a016618 26511629 PMC4665078

[bib17] Hunter N. Borts R. H., 1997 Mlh1 is unique among mismatch repair proteins in its ability to promote crossing-over during meiosis. Genes Dev. 11: 1573–1582. 10.1101/gad.11.12.1573 9203583

[bib18] Jessop, L., and M. Lichten, 2008 Mus81/Mms4 endonuclease and Sgs1 helicase collaborate to ensure proper recombination intermediate metabolism during meiosis. Mol. Cell 3: 313–323. 10.1016/j.molcel.2008.05.021 PMC2584117 18691964

[bib19] Kaur, H., A. De Muyt, and M. Lichten, 2015 Top3-Rmi1 DNA single-strand decatenase is integral to the formation and resolution of meiotic recombination intermediates. Mol. Cell 4: 583–594. 10.1016/j.molcel.2015.01.020 PMC4338413 25699707

[bib20] Keeney S., 2008 Spo11 and the Formation of DNA Double-Strand Breaks in Meiosis. Genome Dyn. Stab. 2: 81–123. 10.1007/7050_2007_026 21927624 PMC3172816

[bib21] Keeney S. Giroux C. N. Kleckner N., 1997 Meiosis-specific DNA double-strand breaks are catalyzed by Spo11, a member of a widely conserved protein family. Cell 88: 375–384. 10.1016/S0092-8674(00)81876-0 9039264

[bib22] Kim S. Peterson S. E. Jasin M. Keeney S., 2016 Mechanisms of germ line genome instability. Semin. Cell Dev. Biol. 54: 177–187. 10.1016/j.semcdb.2016.02.019 26880205

[bib23] Kneitz B. Cohen P. E. Avdievich E. Zhu L. Kane M. F., 2000 MutS homolog 4 localization to meiotic chromosomes is required for chromosome pairing during meiosis in male and female mice. Genes Dev. 14: 1085–1097. 10809667 PMC316572

[bib24] Kolas N. K. Cohen P. E., 2004 Novel and diverse functions of the DNA mismatch repair family in mammalian meiosis and recombination. Cytogenet Genome Res 107: 216–231. 10.1159/000080600 15467367

[bib25] Kolas N. K. Svetlanov A. Lenzi M. L. Macaluso F. P. Lipkin S. M., 2005 Localization of MMR proteins on meiotic chromosomes in mice indicates distinct functions during prophase I. J. Cell Biol. 171: 447–458. 10.1083/jcb.200506170 16260499 PMC2171243

[bib26] Lahiri S. Li Y. Hingorani M. M. Mukerji I., 2018 MutSγ-Induced DNA Conformational Changes Provide Insights into Its Role in Meiotic Recombination. Biophys. J. 115: 2087–2101. 10.1016/j.bpj.2018.10.029 30467025 PMC6289823

[bib27] Lipkin S. M. Moens P. B. Wang V. Lenzi M. Shanmugarajah D., 2002 Meiotic arrest and aneuploidy in MLH3-deficient mice. Nat. Genet. 31: 385–390. 10.1038/ng931 12091911

[bib28] Lynn A. Soucek R. Börner G. V., 2007 ZMM proteins during meiosis: crossover artists at work. Chromosome Res. 15: 591–605. 10.1007/s10577-007-1150-1 17674148

[bib29] Modrich P. Lahue R., 1996 Mismatch repair in replication fidelity, genetic recombination, and cancer biology. Annu. Rev. Biochem. 65: 101–133. 10.1146/annurev.bi.65.070196.000533 8811176

[bib30] Nishant K. T. Chen C. Shinohara M. Shinohara A. Alani E., 2010 Genetic analysis of baker’s yeast Msh4-Msh5 reveals a threshold crossover level for meiotic viability. PLoS Genet. 6: e1001083 10.1371/journal.pgen.1001083 20865162 PMC2928781

[bib31] Nishant K. T. Plys A. A. R. O. N. J. Alani E. R. I. C., 2008 A mutation in the putative MLH3 endonuclease domain confers a defect in both mismatch repair and meiosis in Saccharomyces cerevisiae. Genetics 179: 747–755. 10.1534/genetics.108.086645 18505871 PMC2429871

[bib32] Novak J. E. Ross-Macdonald P. B. Roeder G. S., 2001 The budding yeast Msh4 protein functions in chromosome synapsis and the regulation of crossover distribution. Genetics 158: 1013–1025. 11454751 10.1093/genetics/158.3.1013 PMC1461720

[bib33] Oh S. D. Lao J. P. Taylor A. F. Smith G. R. Hunter N., 2008 RecQ helicase, Sgs1, and XPF family endonuclease, Mus81-Mms4, resolve aberrant joint molecules during meiotic recombination. Mol. Cell 31: 324–336. 10.1016/j.molcel.2008.07.006 18691965 PMC2587322

[bib34] Pattabiraman D. Roelens B. Woglar A. Villeneuve A. M., 2017 Meiotic recombination modulates the structure and dynamics of the synaptonemal complex during C. elegans meiosis. PLoS Genet. 13: e1006670 10.1371/journal.pgen.1006670 28339470 PMC5384771

[bib35] Pochart P. Woltering D. Hollingsworth N. M., 1997 Conserved properties between functionally distinct MutS homologs in yeast. J. Biol. Chem. 272: 30345–30349. 10.1074/jbc.272.48.30345 9374523

[bib36] Robert T. Nore A. Brun C. Maffre C. Crimi B., 2016 a The TopoVIB-Like protein family is required for meiotic DNA double-strand break formation. Science 351: 943–949. 10.1126/science.aad5309 26917764

[bib37] Robert T. Vrielynck N. Mézard C. de Massy B. Grelon M., 2016 b A new light on the meiotic DSB catalytic complex. Semin. Cell Dev. Biol. 54: 165–176. 10.1016/j.semcdb.2016.02.025 26995551

[bib38] Romanienko P. J. Camerini-Otero R. D., 2000 The mouse Spo11 gene is required for meiotic chromosome synapsis. Mol. Cell 6: 975–987. 10.1016/S1097-2765(00)00097-6 11106738

[bib39] Santucci-Darmanin S. Paquis-Flucklinger V., 2003 Les homologues de MutS et de MutL au cours de la méiose chez les mammifères. Med. Sci. (Paris) 19: 85–91. 10.1051/medsci/200319185 12836196

[bib40] Schwartz E. K. Heyer W.-D., 2011 Processing of joint molecule intermediates by structure-selective endonucleases during homologous recombination in eukaryotes. Chromosoma 120: 109–127. 10.1007/s00412-010-0304-7 21369956 PMC3057012

[bib41] Shodhan A. Lukaszewicz A. Novatchkova M. Loidl J., 2014 Msh4 and Msh5 function in SC-independent chiasma formation during the streamlined meiosis of Tetrahymena. Genetics 198: 983–993. 10.1534/genetics.114.169698 25217051 PMC4224184

[bib42] Snowden T. Acharya S. Butz C. Berardini M. Fishel R., 2004 hMSH4-hMSH5 recognizes Holliday Junctions and forms a meiosis-specific sliding clamp that embraces homologous chromosomes. Mol. Cell 15: 437–451. 10.1016/j.molcel.2004.06.040 15304223

[bib43] Snowden T. Shim K.-S. Schmutte C. Acharya S. Fishel R., 2008 hMSH4-hMSH5 adenosine nucleotide processing and interactions with homologous recombination machinery. J. Biol. Chem. 283: 145–154. 10.1074/jbc.M704060200 17977839 PMC2841433

[bib44] Svetlanov A. Baudat F. Cohen P. E. de Massy B., 2008 Distinct functions of MLH3 at recombination hot spots in the mouse. Genetics 178: 1937–1945. 10.1534/genetics.107.084798 18430927 PMC2323788

[bib45] Turner J. M. A. Aprelikova O. Xu X. Wang R. Kim S., 2004 BRCA1, histone H2AX phosphorylation, and male meiotic sex chromosome inactivation. Curr. Biol. 14: 2135–2142. 10.1016/j.cub.2004.11.032 15589157

[bib47] Wang T. F. Kleckner N. Hunter N., 1999 Functional specificity of MutL homologs in yeast: evidence for three Mlh1-based heterocomplexes with distinct roles during meiosis in recombination and mismatch correction. Proc. Natl. Acad. Sci. USA 96: 13914–13919. 10.1073/pnas.96.24.13914 10570173 PMC24165

[bib48] Wiltshire T. Park C. Caldwell K. A. Handel M. A., 1995 Induced premature G2/M-phase transition in pachytene spermatocytes includes events unique to meiosis. Dev. Biol. 169: 557–567. 10.1006/dbio.1995.1169 7781899

[bib49] Yokoo R. Zawadzki K. A. Nabeshima K. Drake M. Arur S., 2012 COSA-1 reveals robust homeostasis and separable licensing and reinforcement steps governing meiotic crossovers. Cell 149: 75–87. 10.1016/j.cell.2012.01.052 22464324 PMC3339199

[bib50] Zalevsky J. MacQueen A. J. Duffy J. B. Kemphues K. J. Villeneuve A. M., 1999 Crossing over during Caenorhabditis elegans meiosis requires a conserved MutS-based pathway that is partially dispensable in budding yeast. Genetics 153: 1271–1283. 10545458 10.1093/genetics/153.3.1271 PMC1460811

